# Sister Olive: A Hospital Romance

**Published:** 1886-12-25

**Authors:** W. J. Eccott


### SISTER OLIVE: A HOSPITAL ROMANCE.

By W. J. ECCOTT.

### CHAPTER V.

A GLANCE at his watch told Grant that he was too late to catch the last train for Fothercliffe. He sent out for a hackney carriage. Meanwhile, he explained to Halsden the circumstances that necessitated his leaving Cottingham at once; and, having talked the matter over with his old friend and agreed upon the best course of action, he provided himself with the most likely drugs, and set off for Fothercliffe, which was some six miles away.

The carriage was soon rumbling over the cobble-stones through the market-place, which was still murkily luminous with naphtha burners, and resounding with the discordant shoutings of the various vendors, then down a few streets of shops, and, afterwards, along the whole monotonous length of three streets of tenement houses, inhabited chiefly by mill hands and factory workers. The noise and glare of the town rendered thinking difficult. But once out into the quiet country roads, Hamelin Grant could ponder on the strange history, and this stranger incident of the evening.

This, then, was the reason why Sister Olive could not listen to his suit. She had conceived it to be her duty to watch over her brother as far as his eccentric movements would permit; to bear alone the grief that his evil courses occasioned, and, in a sense, expiate his wrong-doing by a life of self-sacrifice. It was because she would not link with that life of self-abnegation any other's, as long as her brother remained a cloud hovering on her horizon, a cloud which might spread abroad, and cast a shadow of great darkness in the shape of social disgrace, not only over herself but those whom she might hold dear. She had resolved to wear, herself, the "white flower of a blameless life," but there were thorns bound up inseparably in the same garland. She had been wearing this piercing though the thorns were, all her young life, when, had she been so minded, she might have spurned her brother as a scapegrace and a ne'er-do-well, as many others would have done, and yet retained the esteem of the world. She had given up all the social amenities to which her beauty and position entitled her, and for what?—to make some reparation for the sins that her too-well-loved brother was daily and hourly committing. And now, she had but just returned from her mission of reconciliation, to take up the self-imposed task of watching by the sick-bed of her boy admirer, whose only claims on her were the small attentions that, in the outburst of his young love, he had bestowed upon her. Grant's whole soul thrilled with emotion as he thought of her; a thrill such as passes through our frame when we hear, in every-day life, of some heroic action or suffering. With Grant there was no thought of self now. His thoughts still ran on what Hargen had only hinted at, but the gaps in which he, with his appreciation of her character, could vividly fill in. Then, again, Archie Tenison was recalled to his mind. Hard-working student, Grant knew him to be, and a man who would make a first-class surgeon some day. And, now, he was suddenly struck down by what might prove a most dangerous (Olive had mentioned it as serious) affection; and that, too, in the devoted pursuit of that lore which was to be the weapon with which, in after life, he was to join in the great struggle against Disease and Death.

"After all," thought Grant, "we are two comrades in the same army, and, in the face of such self-sacrifice as Olive's—away with thoughts of mean and petty rivalry. Let me pull him through this illness. Then let him win her if he may. But to save him—that must be done." And, most earnestly, he preferred a silent pleading to the Great Physician to give His help in this crisis. Again, he thought over carefully all he knew on the subject of similar blood poisonings, and had all his mental armoury at hand, when the carriage drew up at "The Birkholts."

After dismissing the driver and entering the hall, he was met by a lady wearing a widow's cap. She was evidently past middle age, but her features had not lost their youthful bloom nor her carriage its elasticity. Coming forward, she held out her hand and drew him aside into a little sitting-room, where the books upon the shelves, chiefly medical books, a tennis racket, a collection of pipes on the mantel, and other signs betokened that it was Archie Tenison's study.

"Doctor Grant, I believe?" she said. "I am Archie's mother. It is very good of you to come so readily. And Sister Olive! such a kind, lovable lady—to come, too. What a blessed thing it is to have such friends in this trouble. Poor Archie! I had no idea, nor had he, this was to come from a trifling accident. But he's ill, very ill, Doctor. And I'm so nervous of anything happening to him. You see, being his mother, and he the only child, and such a good lad he's always been, so hard-working.—Oh! I do hope it's not dangerous. But I know he's in good hands. He's often spoken of Sister Olive; and she'll nurse him so much better than I should. I'm so nervous." Here Mrs. Tenison, despite her attempts to conceal her trouble, nearly broke down. But she went on in a minute or so: "Would you like anything after your drive, before you go up?"

"No, thank you, Mrs. Tenison. You can rely on my doing all I can; and I see you've made friends with Sister Olive already. Kindly show me up at once."

As Mrs. Tenison opened the bedroom door, Olive was sitting at the bedside administering a cooling drink to Archie, who lay prostrate. She rose with a look of great joy as Grant entered, and the grasp of her hand told her thanks for his prompt compliance with her summons.

Archie turned his head as Grant entered, gave a look of inquiry up into his face, then seem satisfied, and lay back as before. "Well, Archie (everyone called him Archie), are you laid up? What's this I hear about you? Tell me how you managed it."

Mrs. Tenison had not stayed. Archie spoke in an anxious, nervous way, breathing somewhat hurriedly:

"I cut my finger up in the ward two days ago. But it didn't bleed much. Then, yesterday morning, I went down to assist Sandy in a post-mortem. I forgot to put a finger-stall on, and must have got my blood poisoned through the cut. Yesterday evening, my finger looked inclined for a whitlow. Then pain began to spread up to my armpits. Last night it increased, and now just look at my temperature!" and he handed Grant a pocket thermometer, with which he had been testing his temperature continually, getting more nervous each time. His face flushed and grew pale by turns, and, although the room was warm, he shivered now and again.

"Dangerous fever isn't it?" he asked in a hoarse tone. "But don't make much of it to mother. It would distress her so—and"—he almost whispered—"she's so fond of me, you know."

"You mustn't give up at the very outset," said Grant cheerily, having noted the indications of the thermometer—"and with such a nurse, too, you're bound to pull through. You've got that gold medal to win, you know. Come, let me look at the wound."

Grant carefully examined it, and saw that the effects of the poison were showing themselves markedly right up the arm. The pulse was beating rapidly. Feverish symptoms had set in unmistakably. Now and again those shiverings recurred, and then those sudden flushes. He carefully re-dressed the hand, and then asked:

"Did you have much sleep last night?"

"Scarcely any," said Archie, turning his head a little to try and read Grant's face.

"And to-day?"

"None! I've been uneasy, hot. Sister Olive, did you put those temperatures down?"

"Yes," said Sister Olive, and handed them to Grant; while Archie went on:

"And I should have been precious miserable if it hadn't been for Sister Olive coming,"—and here a grateful look came over his face.

"You must get some sleep to-night, at all events," said Grant. "I'll mix you a sleeping-draught."

"I shall be glad, I'm sure," murmured Archie. "I'm very tired."

While Grant was mixing the sleeping-draught in an adjoining small room, which opened out of the sick-room, Sister Olive took occasion to look in and whisper:

"What do you think of him? Is it very serious?"

"More so than I expected," said Grant. "The fever's rapidly increasing. He will require the most careful attention and most constant watching—but I can depend on you, Sister Olive, I know. If this fever goes on, after he wakes up from that draught, you must give him a few spoonfuls of strong beef-tea to keep him up. In the meantime go down and get some supper while I am here. But, one minute! You haven't told me how it was you happened to come here?"

"You've heard that I have left the Infirmary? I left Cottingham the same day to visit some relatives, and came back this morning. I happened to call at the Infirmary, and heard from a student who came in as I was talking, that Archie Tenison was ill. I thought there would be no harm in calling to see how he was, and, finding it more serious than I thought, I offered my help to Mrs. Tenison, who was very grateful. Remembering your last request, Dr. Grant, I sent for you."

"You did right!" said Grant earnestly, grasping her hand. "Now, run down to Mrs. Tenison and cheer her up."

(*To be continued.*)

---

The Cause of Death.—The subject being taken in class was heathen mythology. The head governess was questioning the young ladies about Io, the lovely priestess of Juno. "What did Io die of?" asked the mistress. "I do not know," said the cleverest girl in the school, "unless *Io-dide* of potassium." The naughty damsel had a heavy imposition to do that afternoon.

